# A Longitudinal 5-Year Follow-Up Study of Cognitive Function After First Episode Major Depressive Disorder: Exploring State, Scar and Trait Effects

**DOI:** 10.3389/fpsyt.2020.575867

**Published:** 2020-12-07

**Authors:** Eivind Haga Ronold, Marit Therese Schmid, Ketil Joachim Oedegaard, Åsa Hammar

**Affiliations:** ^1^Department of Biological and Medical Psychology, University of Bergen, Bergen, Norway; ^2^Department of Welfare and Participation, Faculty of Health and Social Sciences, Western Norway University of Applied Sciences, Bergen, Norway; ^3^Division of Psychiatry, Haukeland University Hospital, University of Bergen, Bergen, Norway; ^4^Department of Psychiatry, University of Bergen, Bergen, Norway

**Keywords:** major depression and executive dysfunction, first episode major depressive disorder, processing speed, risk factors, rumination, state, trait, scar

## Abstract

Major depression (MDD) is associated with cognitive deficits in processing speed and executive function (EF) following first episode (FE). It is unclear whether deficits are state or trait related. Studies following FE MDD over longer periods are lacking, making it uncertain how cognition and symptoms develop after the initial episode. The present study assessed cognitive function and symptoms 5 years following FE MDD. In addition, the study explored relationships between MDD symptoms, rumination, and cognitive deficits with regards to the trait, state, and scar perspective. Twenty-three participants with previous FE MDD, and 20 matched control participants were compared on Delis-Kaplan Executive Function System measures of processing speed and EF, in a 5-year longitudinal follow-up study. Correlations between current symptoms- and history of MDD, rumination, cognition were investigated. Findings indicated that cognitive deficits persisted with no clear signs of exacerbation after initial episode. Inhibition appeared independent of current and previous symptoms of depression. Processing speed was related to depressive- symptoms and rumination. In conclusion, results indicated persisting, stable deficits in both EFs and processing speed. Findings further suggest that depressive symptoms could be related to deficits in processing speed, indicating state effects. There was limited support for worsening of cognition after initial episode. Some aspects of EF like Inhibition could show persistent deficits independent of depressive symptoms indicating trait effects.

## Introduction

Major Depressive Disorder (MDD) is one of the most prevalent and taxing disorders worldwide (1). Recurrence following first episode MDD are of particular concern (2), with estimate rates up to 90% in health care seeking individuals (3). Recurrence leaves patients with higher disability, lower quality of life, affecting everyday functioning (4, 5), and could be incremental (6, 7). Residual symptoms are important risk factors in recurrent MDD (8). Cognitive deficits persisting in remission (9–11), could contribute to relapse, recurrence, and impaired daily functioning in MDD (12–14). However, the literature is mixed regarding whether deficits in executive- (15–17), or lower cognitive functions persist in remission (11, 18). Hierarchical organization of neuropsychological function implies that lower processing tasks are the foundation of higher cognitive functions like EF (19). Thus, separating EF from processing speed, seems to be important when investigating cognitive deficits following MDD (20, 21). How EF and processing speed relate to other residual symptoms and risk factors in remission from MDD is uncertain (22, 23), however.

Not everyone with MDD shows cognitive deficits (24). Differences in risk factors for cognitive deficits could help explain this, and include: depression status (9, 10, 15, 25), depressive symptoms (26), number- and length of episodes (11, 27), comorbid disorders (19, 20, 28, 29), rumination (30). In addition, comorbidity (31), and rumination [for a recent review see (32)], have been associated with a more severe course of illness. According to Allot et al. (22) development of both MDD and EF occur in parallel in adolescence and early adulthood. Thus, following a group of young adults from FE MDD reduces risk factors and moderators like age (33, 34). Moreover, longitudinal studies investigating FE MDD over longer periods in remission are lacking (23), precluding how cognition develops following FE MDD.

Many central issues regarding the neurocognitive profile in MDD can be illustrated by the state, trait and scar debate [for a discussion see (23, 35)]. *States* can be understood as deficits caused by-, and fluctuating with-, depressive symptoms. There are mixed findings regarding state effects on EF (11, 17, 26). Findings are also mixed regarding processing speed (11, 25), but most authors seem to find a relationship to depressive symptoms or status (15, 26, 36, 37). *Scars* are neurobiological changes due to previous depression or environmental stressors. Scarring could include length, number, and severity of MDD, resulting in exacerbated cognitive impairment (22). Neurobiological changes could also increase risk for further episodes of MDD (7). In addition to this, common treatments could mediate changes and further alter neurocognitive function (25, 38). Scarring effects could be investigated through the relationship between previous MDD duration and symptom severity, and later cognitive function (22). Semkovska et al. (11) found number of episodes negatively influenced attention, processing speed, verbal fluency, and task shifting, supporting scar effects. When manifested as *traits*, impairments are independent of scars and current symptom states, predating FE MDD. There seems to be most agreement on persistent deficits in EFs (15, 25, 36, 39), with mixed findings regarding verbal fluency (15, 25, 36, 39).

The current study was a 5 year longitudinal study, investigating EF, inhibition, working memory/mental flexibility, and verbal fluency, in addition to motor- and processing speed. Previous studies investigating the FE group found deficits in EFs and processing speed in the acute phase- (40), and 1 year following FE MDD (41). (41) found lasting impairments in the EF tasks Inhibition/Switching and verbal fluency. The current study investigated if deficits and symptoms persisted or normalized, after 5-years. In addition, the trait, state, and scar perspective were utilized in exploring the findings. To the authors knowledge, this is the first study to measure cognition in a group with FE MDD over 5 years. Consequently, the study could contribute to an increased understanding of the longitudinal development of cognitive residual symptoms and course of illness following FE MDD. The following hypotheses were investigated:

1) We predict that cognitive deficits persist after 5 years, and that a group with previous FE MDD will differ from a matched control-group, on tests measuring both processing speed and EFs.

2) It is expected that cognitive deficits and rumination related to depressive symptoms at time of assessment could represent state effects. Cognitive deficits and symptoms related previous length and strength of depression, worsening over time, could represent scar effects. Cognitive deficits that are relatively stable and independent of current- and previous symptoms of depression, could represent traits. EF is suspected to be relatively independent of state and scar effects, and a relationship between EF and rumination is expected. Processing- and motor speed are suspected to be influenced by depressive state and scar effects.

## Materials and Methods

### Design

**Figure 1:**
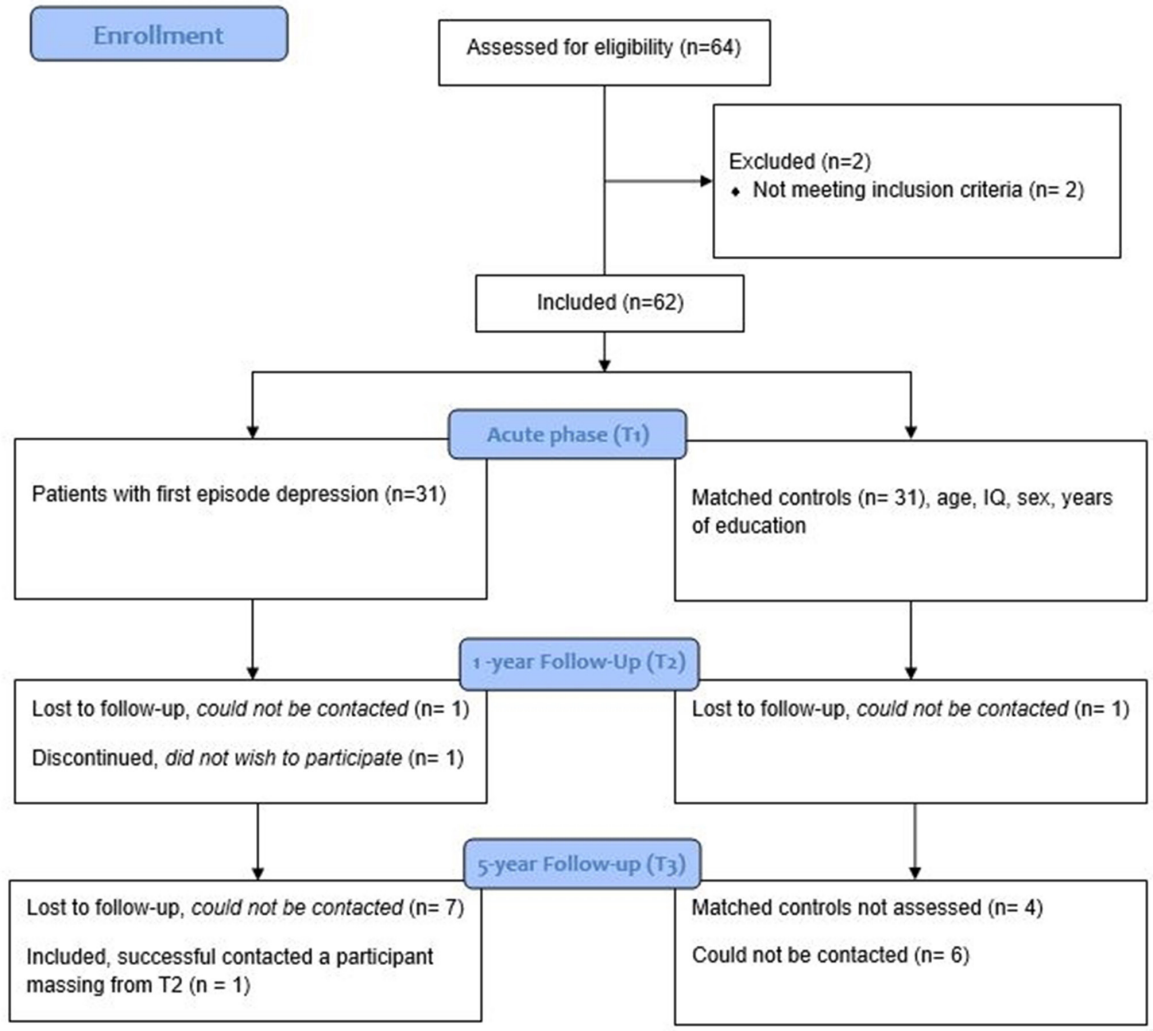
Participant flowchart.

This longitudinal 5-year case control follow-up study examined a group with FE MDD and matched controls. There were three points of assessment: Participants were assessed at baseline in the acute phase of MDD (T1), after 1 year (T2), and after 5 years (T3). For additional information, see Figure 1.

### Recruitment and Participant Flow

Participants in the depression group (DG) were recruited from primary healthcare and student healthcare services in Bergen, Norway. Participants were informed about the ongoing study through the cooperation of physicians and psychologists in primary- and university healthcare clinics. A study coordinator contacted interested patients that met the inclusion criteria at T1. Initial inclusion criteria were current FE depression of a moderate- to severe degree, indicated by a score of ≥20 on the Montgomery Åsberg Depression Rating Scale [MADRS; (42)]. Initial exclusion criteria for the DG were earlier history-, treatment-, or diagnosis of depression. Exclusion criteria for all participants in the DG from T1, T2, and T3 were the following: Psychosis, electroconvulsive treatment, alcohol- or substance abuse, brain damage, neurological- or severe somatic disorder. A trained psychologist assessed exclusion criteria at each time point by a structured questionnaire (T1, T2, and T3), and The Norwegian version of Mini-International Psychiatric structural interview [MINI; (43)] for the DG (T1 and T2). A control group was recruited, individually matched on the following variables: Sex, age, and years of education (±2 years), and the matching was valid at T3 (see Table 1). Controls were recruited at the University of Bergen and through colleges at the Department of Biological and Medical Psychology. Exclusion criteria for controls were: History of any mental disorder, alcohol- or substance abuse, brain damage, neurological- or severe somatic disorder, measured by a structured questionnaire designed for the study (at T1, T2, and T3). All participants were invited to a 1-year follow up assessment (T2) at T1. At the 5-year follow up assessment (T3), participants were contacted by mail and invited to take part in the study. Dropouts were largely participants that the study was unable to contact due to expired contact information, and participants that had moved away (see Figure 1).

**Table 1:** Demographics and clinical measures.

**Demographics and** **clinical measures for** **T3 groups (M/F)**	**Depression group** ***n* = 23 (11/12)**	**Control group** ***n* = 20 (9/11)**
	**M (SD)**	**M (SD)**
Age	30.34 (5.74)	30.45 (6.09)
Education	15.34 (2.34)	16.6 (2.01)
IQ^a^	118.65 (8.47)	119.7 (8.27)
Age of onset MDD	25.61 (5.73)	
T2 RRS^b^	45.49 (11.89*) n* = 22	
T3 RRS^***^	48.43 (13.31)	30.15 (9.74)
T2 RRQ^***^	44.90 (8.99*) n* = 20	30.26 (7.26) *n* = 19
T3 RRQ^***^	42.13 (12.52)	32.40 (8.02)
T1 MADRS^b^	24.43 (3.8)	
T2 MADRS^b^	10.27 (5.64) *n* = 22	
T3 MADRS^b^	8.87 (8.13)	
Months depressed since T2	12.60 (14.45) *n=* 21	

*M, Mean; SD, Standard Deviation; M/F, Males/Females; n, Number of participants; IQ, Intelligence Quotient*.

a measured at inclusion

*** Significantly different between groups p < 0.001, RRS, Ruminative Responses Scale; RRQ, Rumination-Reflection Questionnaire (Rumination subscale); MADRS, Montgomery Aasberg Depression Rating Scale;

b *CG were not assessed*.

### Clinical Assessments and Rumination Measures

Clinical assessments were made by trained psychologists. MINI was used to assess history of psychiatric disorders in the DG at T1 (version 5.0), and at T3 (version 6.0). All in the DG met DSM-IV criteria for MDD as measured by MINI. Depressive symptoms were measured by MADRS at all time points. At T3 a retrospective assessment of relapse-, recurrence, and duration of MDD was done using the National Institute of Mental Health's Life Chart Method [NIMH LCM; (44)]. The LCM was used to measure relapse and asses months of depression in the DG since T2. Relapse was defined as reporting one or more depressive episode since T2. Structured interviews were done in the DG assessing exclusion criteria, the use of psychotropic medication and psychological treatment. Controls were not assessed by clinical interviews or MADRS, as psychiatric disorders were exclusion criteria in this group.

Both the DG and controls completed self-report assessments of depressive- and neurotic rumination at both T2 and T3. Depressive rumination was measured by the Norwegian version of the Ruminative Responses Scale [RRS; (45)], a 22 item four point likert questionnaire that measures ruminative responses to depressive mood. The Norwegian version of the 12 item five point likert rumination subscale of Rumination-Reflection Questionnaire (RRQ) measured rumination independently of depressive mood. This form of self-rumination is associated with the personality trait neuroticism (46, 47). At T2 RRS was only administered to the DG. RRQ was administered in both groups at T2 and T3 (see Table 1).

### Clinical Profile

Participants differed in how their course of illness developed following FE MDD. At T3 symptom severity as measure by MADRS ranged from not depressed <12 (*n* = 16), to mild- 12–22 (*n* = 5), and moderate symptoms 23–29 (*n* = 2) as indicated by the Norwegian MADRS manual (48). Seventeen patients had one or more episodes of relapse (74%) between T2 and T3. Almost half of the group (*n* = 11) had histories of comorbid psychiatric symptoms as assessed by MINI at T1 and T3, hereunder: Generalized Anxiety Disorder, Panic Disorder With- and Without Agoraphobia, Agoraphobia, Obsessive Compulsive Disorder, and Social Phobia. There were no instances of exclusion criteria as assessed by MINI; neither Psychotic Disorder nor Alcohol- or Substance Abuse. At T3 four patients were using psychotropic medication: One was currently using a Selective Serotonin Reuptake Inhibitor medication (SSRI: Escitalopram), while two were using Selective Serotonin-Noradrenalin Reuptake Inhibitors (SNRI: Venlafaxine). Finally, one participant was prescribed a sedative (Chlorprothixene: Truxal). The same patients were also receiving psychological treatment and outpatient psychiatric follow up. One patient was currently in psychotherapy, while three were in contact with the District Psychiatric Center for outpatient follow up of psychotropic mediation and/or psychotherapy (see Table 1 for more clinical characteristics).

### Ethics and Compensation

All participants gave informed consent to participate in the study. Participants received a gift card valued at 400 Norwegian Kroner (~50 United States Dollars) for their participation. The study was approved by the Regional Committee for Medical and Health Research Ethics and was performed in accordance with the World Medical Associations Declaration of Helsinki regarding Ethical Principles for Medical Research Involving Human Subjects (49).

### Neuropsychological Assessment

Neuropsychological testing took place at the University outpatient clinic for neuropsychology at all points of assessment. Experienced test technicians did the neuropsychological assessment. IQ was measured at inclusion (T1) by Wechsler Abbreviated Scale of Intelligence [WASI; (50)]. Participants completed a battery of standardized neuropsychological tests at all time points. EF and processing speed were assessed by Delis et al. (51) Delis-Kaplan Executive Function System (D-KEFS). Three subtests from the battery were investigated: The Color Word Interference Test (CWIT) measuring processing speed, inhibition, switching and general EF (21), the Verbal Fluency Test (VFT), and the Trail Making Test (TMT) measuring motor- and processing speed, as well as switching (52). See Supplementary Materials 1 for description of tasks.

Contrast-, composite-, and error scores were computed based on descriptions in the D-KEFS manual (51), and previous studies (41). Contrast scores were made to separate EFs from processing speed, subtracting the Color Naming and Word Reading conditions from the Inhibition- [Inhibition Contrast = Inhibition – (Color Naming + Word Reading)/2], and Inhibition/Switching conditions [Inhibition/Switching Contrast = Inhibition/Switching – (Color Naming + Word Reading)/2]. An EF composite score was for the CWIT overall by separating the processing speed- from the EF conditions [CWIT Executive function composite = (Inhibition + Inhibition/Switching)/2 – (Color Naming + Word Reading)/2]. To separate processing speed and lower cognitive processes from EF, contrast scores for TMT Number-Letter Switching were calculated [Contrast Number-Letter Switching = Number-Letter Switching – (Visual Scanning + Letter Sequencing + Number Sequencing + Motor Speed)/4]. Error scores were pooled for the different D-KEFS tests. In addition, CWIT error scores were pooled to represent executive dysfunction in the Inhibition and Inhibition/Switching conditions, and processing deficits in the Color Naming and Word Reading conditions.

### Data Scoring and Analysis

All statistical analyses were performed in Statistical Package for the Social Sciences (SPSS version 25). Raw scores, that consisted of seconds to complete task (CWIT, TMT, high score = poor performance), and words generated per minute (VFT, high score = good performance), were used for the neuropsychological tests. Variables were plotted and checked for linearity and outliers. Outliers were inspected and determined to represent real scores and not errors. Normality was assessed using Kolmogorov-Smirnov test of normality and non-parametric tests were used when assumptions were violated. Cohen (53) was used to describe effect sizes as small, medium and large.

#### Differences Between the DG and Controls in Cognitive Function Over Time

Mixed between-within subjects ANOVA was used to calculate differences between groups in cognitive function and change over the three points of assessment (Group × Time × D-KEFS conditions). Box's test of equality of Error Variance and Mauchly's Test of Sphericity was performed and Multivariate statistics reported when the latter assumption was violated. Levenes test of Equality of Error Variances was performed for all ANOVA analyses, and Welch values given when this assumption is violated. One participant in the DG was only available at T1 and T3 and was thus missing from the Mixed between-within subjects ANOVA, and other analyses containing data from T2 (see Figure 1). Groups at T3 were compared by one-way analysis of variance (ANOVA) on matched variables and clinical measures. Mann Whitney *U*-tests were used to assess differences between groups on non-parametric data, and independent samples chi square tested for categorical variables. Change scores were calculated by subtracting D-KEFS scores at T2 from D-KEFS scores at T3. Negative values implied decreased performance over time (with the exception of VFT, were the opposite was the case). Paired sample *t*-tests were used to assess changes on D-KEFS by comparing scores from T2 to scores from T3 in the DG, and the control group.

#### Separating EF and Processing Speed

One-way ANOVA analyses were used to investigate differences between groups on the different D-KEFS conditions and contrast scores at T3. Man Whitney *U*-tests were used to assess error scores and the CWIT EF composite score. Effect size measures (η^2^) for Man Whitney *U*-tests were calculated through the following formula (η^2^ = Z^2^/N-1).

#### Traits, States, and Scars, Relationships Between Depression, Rumination, and Cognitive Function

Bivariate correlation coefficients were calculated to explore relationships at T3. The relationship between symptoms of MADRS, RRS, RRQ, and D-KEFS scores was investigated. Spearman's Rho was used as a non-parametric alternative to Pearson's correlation coefficients when assumptions for the latter were not met. To separate depressive state (MADRS T3) from scar effects, an explorative composite score consisting of a standardization (Computed in SPSS) of number of months depressed (*Z*-score months depressed), combined with standardized MADRS scores *before* T3 (T1, T2) were calculated: Scar composite = (ZMADRS T1 + ZMADRS T2 + Zmonths depressed)/3.

## Results

### Matching of Groups

Groups did not differ significantly (*p* > 0.05) on any of the matched variables sex, age, and years of education, nor in IQ (see Table 1 for means and frequencies).

### Differences Between the DG and Controls in Cognitive Function Over Time

Mixed between-within subjects ANOVA found a significant interaction effect for Time × Condition in all D-KEFS tests (see Table 2). Means indicated that scores from T1 improved. A lack of a Time × Condition × Group interaction supported that improvements did not differ between groups, and that both processing speed and EF improved similarly over time. In addition, there was a significant main effect of group on the CWIT and TMT, while the VFT only approached significance, with a medium effect size. Overall, groups showed similar improvements, but differed in test performance.

**Table 2:** Cognitive differences between groups over time.

			**Main effects**			**Interaction effects**		
		**Group**	**Condition**	**Time**	**Time × group**	**Time × condition**	**Condition × group**	**Time × cond. × group**
CWIT	Wilk's λ		0.035	0.501	0.918	0.594	0.869	0.910
	*F*_(df)_	13.55 (1, 40)	347.96 (3, 38)	19.34 (2, 39)	1.75 (2, 39)	3.99 (6, 35)	1.906 (3, 38)	0.58 (6, 35)
	Partial eta-sq.	0.253	0.965	0.131	0.082	0.406	0.131	0.090
	F-sig.	*p* = 0.001	*p* < 0.001	*p* < 0.001	*p* = 0.188	*p* = 0.004	*p* = 0.131	*p* = 0.74
VFT	Wilk's λ		0.035	0.772	0.968	0.759	0.918	0.971
	*F*_(df)_	3.48 (1, 40)	534.53 (2, 39)	5.57 (2, 39)	0.653 (2, 39)	2.93 (4, 37)	1.751 (2, 39)	0.28 (4, 37)
	Partial eta-sq.	0.08	0.965	0.228	0.032	0.241	0.082	0.029
	F-sig.	*p* = 0.069	*p* < 0.001	*p* = 0.006	*p* = 0.526	*p* < 0.05	*p* = 0.19	*p* = 0.89
TMT	Wilk's λ		0.085	0.559	0.953	0.446	0.791	0.898
	*F*_(df)_	6.53 (1, 40)	99.53 (4, 37)	15.41 (4, 39)	0.953 (2, 39)	5.12 (8, 33)	2.45 (4, 37)	0.469 (8, 33)
	Partial eta-sq.	0.140	0.915	0.441	0.047	0.554	0.209	0.102
	F-sig.	*p* = 0.015	*p* < 0.001	*p* < 0.001	*p* = 0.39	*p* < 0.001	*p* = 0.063	*p* = 0.87

*CWIT, Color Word Interference Test; VFT, Verbal Fluency Test; TMT, Trail Making Test; (df), degrees of freedom; Partial Eta-sq., Partial Eta Squared*.

One-way ANOVA tests indicated no significant differences between groups on change scores from T2 to T3. This was supported by paired sample *t*-tests on D-KEFS scores from T2 to T3, that showed there were no significant improvements in scores, with the exception of controls improving on TMT Number Sequencing from T2 (see Table 3). In sum, this supported that the Time × Condition interaction above was due to changes from T1 to T2, not from T2 to T3. Cognitive deficits persisted after 5 years and were relatively stable following the acute phase of FE MDD.

**Table 3:** Cognitve performance at T3.

**Groups at T3 (M/F)**	**Depression group** ***n*** **=** **23 (11/12)**		**Control group** ***n*** **=** **20 (9/11)**		**Statistics**		
**D-KEFS measure**	**M (SD)**	**Change score from T2 *n* = 22**	**M (SD)**	**Change score from T2**	***F*_**(1, 41)**_**	***p***	**eta sq**.
Color word interference test							
Color naming	29.87 (4.68)	−0.55	26 (4.63)	0.5	^**^7.37	<0.01	0.152
Word reading	22.52 (3.3)	0.5	18.9 (2.43)	0.95	^***^16.37	<0.000	0.285
Inhibition	48.04 (8.28)	−0.95	41 (5.91)	0.1	^***^10.02	<0.001	0.196
Inhibition/switching	56.13 (7.52)	−0.41	48.65 (9.58)	−1.05	^***^8.22	<0.001	0.167
Inhibition contrast	21.85 (6.38)	−0.52	18.55 (5.57)	−0.625	3.13	0.084	0.071
Inhibition/switching contrast	29.93 (5.64)	−0.52	26.2 (8.29)	−1.78	3.05	0.088	0.069
Verbal fluency							
Letter fluency^hs^	50.78 (11.48)	−1.27	55.2 (10.51)	−0.4	1.71	0.198	0.040
Category fluency^hs^	48.48 (10.18)	0.27	51.75 (9.68)	3.1	1.16	0.288	0.027
Category switching^hs^	14.96 (2.2)	0.32	15.4 (2.32)	0.65	0.41	0.527	0.010
Trail making test							
Visual scanning	17.91 (4.5)	0.5	15.25 (2.88)	1	*5.15	0.029	0.112
Number sequencing	24.04 (8.47)	1.95	17.3 (4.92)	2.55^*^	^**^9.78	0.003	0.193
Letter sequencing	24.17 (8.95)	−1.5	17.2 (3.37)	2.55	^**^10.78	0.002	0.203
Number letter switching	62.96 (18.95)	0.045	50.1 (15.26)	2.65	^*^5.88	0.02	0.126
Motor speed	20.13 (9.68)	−0.86	16.8 (5.02)	1.45	1.91	0.174	0.045
Contrast number letter switching	41.39 (15.33)	0.02	33.46 (14.43)	0.76	3.02	0.09	0.069

M/F, Males/Females; n, Number of participants; M, Mean (seconds to complete task, except for verbal fluency); SD, Standard Deviation; Df, Degrees of freedom

* *p < 0.05*,

** *p < 0.01*,

*** *p < 0.001*,

hs *high score indicate high performance in these conditions (words generated)*.

One-way ANOVA tests investigated which of the cognitive functions measured by D-KEFS, processing speed or EF, showed largest differences between groups (see Table 3). The DG performed significantly poorer than controls in all the conditions of the CWIT at T3. The contrast scores for Inhibition and Inhibition/Switching were not significantly different, although differences showed moderate effect sizes. There were significant differences in all the conditions of the TMT, except Motor Speed. The Number-Letter Switching contrast score approached significance, with a moderate effect size. There were no significant differences in the VFT conditions with small effect sizes. Groups significantly differed in conditions measuring both EF and processing speed, but not motor speed. Contrast scores were not significantly different.

### Separating EF and Processing Speed

A significant difference between the groups was found on a composite score for Inhibition and Inhibition/Switching, with the DG performing poorer (*M* = 25.89, *Mdn* = 27.5, *n* = 23) than controls (*M* = 22.38 *Mdn* = 22.75, *n* = 20) *U* = 146.5, *p* = 0.023, η^2^ = 0.099. Error scores from the two processing speed conditions were also compared to the executive conditions. Errors in the processing speed conditions differed with the DG making more errors (*M* = 0.87, *Mdn* = 1), and controls fewer (*M* = 0.25, *Mdn* = 0), Mann Whitney *U* = 150.5, *p* = 0.023, η^2^ = 0.123. The DG made more errors in the executive conditions (*M* = 2.13, *Mdn* = 2), compared to controls (*M* = 0.85, *Mdn* = 0.50), Mann Whitney *U* = 92, *p* = 0.001, η^2^ = 0.286. No significant differences in error scores appeared in either VFT or TMT. Differences in composite score and errors in the EF conditions of CWIT, supported deficits in EF when controlling for processing speed.

### Course of Illness

Overall, symptoms of depression and rumination were relatively stable in the DG after T1 (see Table 1). The standard deviation of MADRS and months depressed at T3 suggested increased variance of depression in the DG, however. This could reflect a polarization of depressive symptoms in the group. Comorbid disorders increased in the group. At T1 only (16%) had a history of comorbid disorders (one of these dropped out). At T3 (48%) had a history of comorbid disorders. McNemar's test showed that increased comorbidity was significantly greater than chance from T1 to T3, *p* = 0.039 (2-sided), in the DG. This could indicate considerable heterogeneity regarding course of illness after T1.

### Relationships Between Symptoms and Cognitive Function

Spearman's Rho was calculated to explore the relationship between depressive symptoms and D-KEFS scores at T3 (see Table 4). MADRS showed small to large correlations with CWIT scores. Word Reading showed the largest relationship to MADRS. Relationships between EF (CWIT contrast and composite scores) and T3 MADRS score was small. There were small to medium relationships between CWIT and depressive rumination, showing similar, but smaller relationships than MADRS. MADRS showed a large relationship to depressive rumination supporting this. The CWIT processing speed measures showed small correlations to neurotic rumination. RRQ showed a moderate relationship to Inhibition/Switching, *r* = 0.34 *n* = 23 *p* = 0.112 (2-sided), although this relationship was smaller on the contrast score *r* = 0.303 *n* = 23 *p* = 0.116 (2-sided). CWIT processing speed measures showed the largest relationships to depressive- symptoms and rumination. Neurotic rumination differed, showing only a moderate relationship to Inhibition/Switching.

**Table 4:** Relationships between depressive symptoms, rumination, and cognitive tests.

	**Relationships between symptoms and cognitive tests**	**1**	**2**	**3**	**4**	**5**	**6**	**7**	**8**	**9**	**10**	**11**	**12**	**13**	**14**	**15**	**16**	**17**	**18**
1	MADRS	1																	
2	RRS	0.541^**^	1																
3	RRQ	0.305	0.810^**^	1															
4	CWIT: color naming	0.341	0.174	0.141	1														
5	CWIT: word reading	0.528^**^	0.426^*^	0.155	0.512^*^	1													
6	CWIT: inhibition	0.049	0.037	0.217	0.671^**^	0.313	1												
7	CWIT: inhibition/switching	0.363	0.241	0.242	0.656^**^	0.581^**^	0.357	1											
8	CWIT: inhibition contrast	−0.164	−0.076	0.223	0.398	−0.003	0.877^**^	0.014	1										
9	CWIT: Inh/switching contrast	0.182	0.104	0.169	0.266	0.222	0.044	0.847^**^	−0.224	1									
10	VFT: letter fluency	−0.063	0.203	0.252	0.135	−0.236	0.147	0.09	0.227	0.152	1								
11	VFT: category fluency	−0.424^*^	−0.362	−0.101	−0.022	−0.500^*^	0.058	−0.02	0.204	0.09	0.578^**^	1							
12	VFT: category switching	−0.26	−0.231	0.164	0.03	−0.285	0.35	−0.127	0.518^*^	−0.159	0.144	0.591^**^	1						
13	TMT: visual scanning	0.551^**^	0.137	0.053	0.548^**^	0.464^*^	0.388	0.649^**^	0.088	0.430^*^	−0.243	−0.247	−0.168	1					
14	TMT: number sequencing	0.521^*^	0.264	0.088	0.509^*^	0.457^*^	0.335	0.591^**^	0.032	0.420^*^	0.183	−0.121	−0.167	0.667^**^	1				
15	TMT: letter sequencing	0.545^**^	0.171	0.034	0.560^**^	0.387	0.15	0.443^*^	−0.025	0.199	0.002	−0.029	0.119	0.550^**^	0.457^*^	1			
16	TMT: number-letter switching	0.489^*^	−0.088	−0.246	0.580^**^	0.244	0.331	0.376	0.1	0.221	0.055	0.025	0.024	0.538^**^	0.662^**^	0.681^**^	1		
17	TMT: motor speed	0.239	0.096	0.009	0.369	0.539^**^	0.178	0.550^**^	−0.165	0.434^*^	−0.292	−0.275	−0.357	0.470^*^	0.402	0.186	0.31	1	
18	TMT: N-L switching contrast	0.308	−0.192	−0.344	0.487^*^	0.09	0.301	0.157	0.154	0.031	0.079	0.097	0.083	0.34	0.472^*^	0.566^**^	0.946^**^	0.124	1

* *p < 0.05 (2-sided)*,

** *p < 0.01 (2-sided), MADRS, Montgomery Aasberg Depression Rating Scale; RRS, Ruminative Responses Scale; RRQ, Rumination-Reflection Questionnaire (Rumination subscale); CWIT, Color word interference test; VFT, Verbal Fluency Test; TMT, Trail Making Test*.

*Bold values represent potential state effects*.

### State Effects VFT and TMT

T3 MADRS showed small to medium relationships to VFT. Category Fluency showed the largest correlation (see Table 4). There were similar but smaller relationships to RRS, and only small relationships to neurotic rumination. MADRS showed small to large correlations with TMT. Most of the processing speed measures showed large relationships to MADRS. TMT Letter Sequencing showed the largest association with MADRS. TMT showed mostly small relationships to both types of rumination. One exception was for the TMT Number-Letter Switching contrast score, that showed a negative moderate relationship with RRQ *r* = −0.3 *n* = 23 *p* = 0.108 (2-sided). Category fluency and the processing speed measures of TMT showed strongest relationships to depressive symptoms. Neurotic rumination showed a moderate negative relationship to Number-Letter Switching contrast score.

### Scar and Trait Effects

The scar composite score was used to investigate how previous history of depression related to D-KEFS scores and rumination at T3. The scar composite showed small correlations to all of the CWIT scores except for the CWIT Inhibition/Switching condition, with a moderate correlation *r* = 0.439 *n* = 23*, p* = 0.036 (2-sided), and an even stronger relationship to the Inhibition/Switching contrast score *r* = 0.486 *n* = 23*, p* = 0.019 (2-sided), indicating that this relationship could not be explained by processing speed. The scar composite showed a large correlation to RRQ at T3 *r* = 0.562, *n* = 23*, p* = 0.005 (2-sided), higher than to current depressive symptoms (see Table 4), which could suggest that neurotic rumination show larger relationship to MDD over time than current depressive symptoms. Differences between the DG and controls in CWIT error scores were new, and could thus represent a scar effect. Error scores on the CWIT, especially errors in the EF conditions, showed weak relationships to the scar composite, *rho* = −0.064 *n* = 23*, p* = 0.773 (2-sided) in addition to MADRS at T3 *rho* = −0.073 *n* = 23*, p* = 0.742 (2-sided), however. The scar composite was related to Inhibition/Switching and neurotic rumination at T3, but not new differences between groups CWIT error scores.

### Traits, Stable Differences Between DG and Controls That Are Unrelated to State and Scar Measures

The CWIT Inhibition contrast score showed small correlations to both state*- rho* = −0.164, *n* = 23*, p* = 0.454 (2-sided), and scar measures of depression, *rho* = −0.069 *n* = 23*, p* = 0.753 (2-sided). It did, however, show a medium correlation to error scores in the executive condition, *rho* = 0.451, *n* = 23*, p* = 0.031, thus EF errors could be related to a trait EF/Inhibition impairment independent of state- and scar effects. CWIT Inhibition score differed between the DG and controls at both T1, T2 and T3, and show negligible relationships to both the scar composite *r* = 0.059 *n* = 23*, p* = 0.788, and MADRS at T3 *rho* = 0.049 *n* = 23*, p* = 0.825 (2-sided), and a comparable relationship to error scores in the EF conditions *rho* = 0.382 *n* = 23*, p* = 0.072 (2-sided). In conclusion, there is some support for stable deficits in CWIT Inhibition that could be independent of state- and scar effects, which thus could represent a cognitive trait deficit in MDD.

## Discussion

The main aim of the present study was to investigate cognitive residual symptoms in the first longitudinal 5-year follow up study of FE MDD. In addition, relationships between current- and previous depression, current rumination, and cognitive deficits were also explored. It was expected that stable deficits, unrelated to current and previous MDD history, could represent traits.

### Persisting Deficits

The first hypothesis predicted that cognitive deficits would persist after 5 years. This hypothesis was supported. Results suggested that there are broad, relatively stable deficits on most of the cognitive measures. Stable differences are in line with several reviews and meta-analyses showing that cognitive deficits persist in remission (9, 11, 15, 36). However, this is the first study to show deficits 5 years following FE MDD. Importantly, there were no indication of significant cognitive decline after initial episode, and therefore little support for a worsening of cognition during the 5 years, although the study could be underpowered to detect small changes.

### Deficits in EF and Processing Speed

Current findings support cognitive deficits in *both* EF and processing speed, in line with the first hypothesis. Although there were larger effect sizes for differences in the latter, there was also moderate effects for differences in EF contrast scores controlling for processing speed. The lack of significant differences in motor speed, also suggest that motor slowing is not sufficient to explain differences on the tests (16, 21). Persisting deficits in EF, even when controlling for processing speed, is mostly supported. This could be contrary to Semkovska et al. (11), were the authors suggest that executive dysfunction is due to deficits in processing speed. Deficits in processing speed showed the largest effects, however.

### Course of Illness and Cognition

Table 1 indicated that the DG differed in their rate of depression following first episode (large standard deviations), which could influence results. In addition, the increase in comorbidity could influence cognitive function and has been shown to have a relationship to processing speed (20), but not inhibition (54). Of note, there was no indication of significant worsening in cognitive functions after T1, and therefore limited evidence to support cognitive exacerbation from the increase in comorbidity. The study could be underpowered to detect this, however. The increase could illustrate the need for longer follow up times in clinical studies, as comorbidity commonly increase with increasing follow up time (31). The relatively high depressive rumination could indicate that rumination represent risk factor in remission from MDD (32, 55, 56). Residual symptoms like rumination, and risk factors like comorbid disorders, could thus be of importance when planning treatment and prevention strategies. The majority of patients had undergone psychological- and/or pharmaceutical treatments, that could have influenced neurocognitive function (25, 38). However, some authors have suggested that at least some cognitive deficits persist despite “successful” treatment (39), as indicated the current study, and thus new interventions targeting cognitive functions seem warranted.

### Are Deficits Associated With Depressive Symptoms States, Scars, or Traits?

There was mixed support for the hypothesized state, scar, and trait effects. Preliminary results supported the hypothesis that processing speed deficits are influenced by state effects. In addition, this finding is in accordance with meta-analyses and reviews showing relationships between MDD and processing speed deficits (26, 37). Similar, albeit weaker, relationships were found between depressive symptoms, depressive rumination, and cognitive tests. This could suggest that the relationship between depressive rumination and cognitive function is due to depressive symptoms in the current sample. The small relationships between depressive rumination and EF went contrary to our expectations. Recent meta-analyses, however, support small relationships between EF and rumination (57–59). Of note, neurotic rumination at T3 showed different associations to cognitive tests, and stronger relationships to EF, compared to depressive rumination. Inhibition and switching were related to neurotic rumination, in support of our hypothesis. Switching, an EF, somewhat unexpectedly showed a moderate negative relationship to neurotic rumination, although some studies have found relationships between rumination and better scores on some aspects of EF (60). Alternatively, this could be a spurious relationship. In conclusion, measuring different forms of rumination (61), like neurotic rumination, could probably further elucidate on the relationship between rumination and cognition.

The exploratory scar composite score showed a relationship to Inhibition/Switching. There was no relationship between the scar composite and contrast score for Inhibition. This could indicate a scar effect on mental flexibility, although this finding must be taken with caution, as there was no clear indication for a worsening of cognitive functions over time. Thus, there is limited support for the scar hypothesis in the present study. Age and follow-up time could explain this however: 5-years might be too short, and participants to young, for a scaring effect to appear. Neuropsychological exacerbation caused by depression could probably be more apparent with increasing age (33). Semkovska et al. (11) found evidence for exacerbation of cognition with number of depressive episodes, although this finding could be influenced by age as well. However, it is hard to conclude about scar effects without measuring cognitive functions before onset of FE MDD.

Of note, neurotic rumination correlated with both the scar composite and Inhibition/Switching. Surprisingly, the relationship between neurotic rumination and history of depression, was higher than that to current depressive symptoms. This could suggest that neurotic rumination is a risk factor for-, or at least associated with MDD history. It could be that neuroticism/rumination and Inhibition/Switching are a part of risk factors for MDD over time. The former could support emerging perspectives for understanding mental illness that focus on neuroticism like the p-factor model (62), while the latter is supported by Schmid and Hammar (41), that found relationships between Inhibition/Switching and relapse and recurrence in a FE sample. Differences between the DG and controls in CWIT error scores are new and could thus represent a scar effect, but was not related to the scar composite. The scar composite is a novel construct based on theoretical assumptions [see Figure 1 in (22)], and might imperfectly capture the nature of the depressive history in our sample, however. In conclusion, there is insufficient evidence in the current paper to conclude regarding the scar hypothesis. Finally, neurotic rumination and Inhibition/Switching could be related to history of depression.

Inhibition, although significantly different between the DG and controls during the 5 years, did not show any sizable or significant relationships to history- or symptoms of depression nor rumination. All this could indicate that inhibition represent a trait and a cognitive risk factor in a group with recurrent depression. The EF function of Inhibition is recognized in other longitudinal studies as a stable deficit in MDD (63–65), in addition to several meta-analyses (9, 17, 36, 37). Inhibition is also the function most strongly associated with the unity EF factor (66), which could point toward a general persisting EF impairment as a trait associated with recurrent MDD.

### Strengths and Limitations

This was the first study to investigate cognition in FE MDD after 5 years. Thus, the study could contribute with a unique perspective on the development of MDD. The current study is important due to the considerable length of follow-up time making it able to assess change and stability in cognitive function in relation to symptoms and course of illness. The thorough neuropsychological testing enabled differentiation between processing speed and EF. In addition, symptoms were measured at different time points, making it possible to investigate longitudinal relationships between cognition and symptoms. Furthermore, the study points to several variables which are relevant for further research, like persisting cognitive deficits (neurotic) rumination, and increased rate of comorbidity following the FE MDD. The increase in comorbidity, however, entails that the study did not asses MDD alone, but also comorbid disorders. This might more accurately reflect the common courses of illness and thus enhance the ecological validity of the current study, but also potentially confound results as discussed above and below. Future studies with larger samples should investigate how risk factors like comorbidity, rumination, relapse, and different treatments mediate and moderate cognition and course of illness in MDD.

Despite some strengths, the study also had major limitations. The results were from a small sample, and a selected group. All participants in the DG were outpatients. IQ was in the average to above average range which could mask deficits. However, the DG and controls showed comparable IQ scores and the groups did not differ on matched variables. MINI does not measure personality pathology (other than antisocial personality disorder) which could have been present- and influenced results. In addition, comorbidity, depressive symptoms, and treatment effects, could have confounded results. Dropout was also considerable. Interestingly, dropout was higher in the control group, which could suggest that clinically unrelated factors played a part in this. Many participants were students that moved away after completing university. The lack of clinical assessments of the controls could be viewed as a major limitation. Symptoms in the control group could have influenced results, but the relatively low rumination scores at T3 (see Table 1) suggested that this was not a major issue, however. There were also issues regarding measurements and sample size. Given the long follow up time, the assessments of months depressed could be influenced by subjective memory and is probably not completely accurate, which could have influenced the novel scar composite. Due to increased type II error rate Bonferroni adjustments were not made to significance levels. This could have increased false positive findings. In addition, the study could be underpowered to detect small changes over the 5 years, which could have resulted in the lack of support for scarring effects. In addition, correlations and other effect sizes might be unstable and inflated due to the small sample (67), and should be interpreted with caution. Results should be considered preliminary and should be replicated in larger samples [for a discussion see (68)]. Also, importantly, correlation does not imply causation, thus the current study cannot say anything about the direction of the relationship between symptoms and cognition. Future studies should longitudinally investigate risk factors in larger samples, making it possible to use more complex statistics to causally model relationships between variables, like in structural equation models. Furthermore, several measures of EFs should be included to facilitate composite scores to more accurately capture the diversity functions of EF and their relationships to symptoms, risk factors, and treatments. Finally, to best inform on the state, trait, and scar debate, prospective longitudinal studies should be done, measuring cognition before the onset of MDD, and thus asses predisposing traits, and potentially scarring effects of FE MDD not captured by the current study.

## Conclusion

The present study indicated that a group of former FE MDD patients showed lasting, stable, deficits in cognition compared to a healthy matched control group after 5 years. There were deficits in both processing speed and EF. Findings suggest that processing speed are related to depressive symptoms indicating state effects. There was no clear worsening of cognitive function. Some aspects of EF like Inhibition showed persistent deficits independent of depressive symptom state, indicating trait effects. The study underscores the importance of persisting cognitive residual symptoms following FE MDD, and the need to adapt treatments and prevention strategies targeting cognitive functioning.

## Data Availability Statement

The raw data supporting the conclusions of this article will be made available by the authors, without undue reservation.

## Ethics Statement

The studies involving human participants were reviewed and approved by the Regional Committee for Medical Research Ethics and the Norwegian Data Inspectorate approved the study. The patients/participants provided their written informed consent to participate in this study.

## Author Contributions

ER: collection and analysis of data, writing manuscript draft, tables, and figures. KO: writing and editing. ÅH: study PI, writing and editing. All authors contributed to the article and approved the submitted version.

## Conflict of Interest

The authors declare that the research was conducted in the absence of any commercial or financial relationships that could be construed as a potential conflict of interest.
