# Supplementary material for: A Longitudinal 5-Year Follow-Up Study of Cognitive Function After First Episode Major Depressive Disorder: Exploring State, Scar and Trait Effects
Source: Front Psychiatry. 2020 Dec 7;11:575867. doi: 10.3389/fpsyt.2020.575867 (PMC7750430; doi:10.3389/fpsyt.2020.575867)
Supplement: Supplementary file 1 [file Data_Sheet_1.docx]

Supplementary materials: Description of D-kefs tests

The D-KEFS CWIT included four different tasks where participants named colors as fast as possible. The two first conditions required the naming of red-, green-, and blue patches (Color Naming)*,* and to read written words of these colors written in black ink (Word Reading). The third condition involved naming the color of the printed letters of an incongruent color word (Inhibition). Participants inhibited the automatic response of *reading* the colored word, and instead named the incongruent color of the ink the word is printed in. In the last condition, participants switched between word reading and inhibition (Inhibition/Switching). The two first conditions are considered measures of processing speed regarding naming and reading. In addition Lezak (2012) described that the word reading- and color naming conditions are sensitive to attentional deficits. The two latter conditions measure EFs like inhibition and switching. Inhibition requires suppression of the automatic tendency to read words. Inhibition/Switching requires switching between reading and suppression, and is understood as a measure of both mental flexibility and inhibition.

The D-KEFS VFT consists of three tasks where participants were instructed to name as many words possible in a one minute interval. There were three conditions: Production of words from specific letters like “S” (Letter Fluency), producing words from categories like “animals” (Category Fluency)*,* and switching between words from two different categories (Category Switching). All of the conditions measure word generating ability, processing speed, attention, retrieval, and simultaneous processing, and verbal ability (Lezak, 2012). Category Switching is considered a measure of mental flexibility in addition to the abilities listed above (Delis et al., 2001)*.*

The D-KEFS TMT consisted of five conditions: Symbol searching (Visual Scanning), connecting of sequential circles based on letters (Letter Sequencing)-, numbers (Number Sequencing)-, and lines (Motor Speed). This is usually understood as a test of visual scanning-, attention-, processing- and motor speed. Furthermore, a condition where alphabetical and numerical circles are connected in an increasing fashion (Number-Letter Switching), has been associated with working memory and mental flexibility which both are considered EFs (Lezak, 2012; Snyder et al., 2015).
